# Silencing protein kinase C ζ by microRNA-25-5p activates AMPK signaling and inhibits colorectal cancer cell proliferation

**DOI:** 10.18632/oncotarget.18649

**Published:** 2017-06-27

**Authors:** Shihu Zhang, Yiyang Zhang, Qing Cheng, Zhaoqun Ma, Guanwen Gong, Zhengming Deng, Kun Xu, Gaoyuan Wang, Yousong Wei, Xiaoping Zou

**Affiliations:** ^1^ Department of General Surgery, Affiliated Hospital of Nanjing University of Chinese Medicine, Nanjing, China; ^2^ Digestive Department, Affiliated Drum Tower Clinical Medical School of Nanjing Medical University, Nanjing, China; ^3^ Department of Gynaecology and Obstetrics, Obstetrics and Gynecology Hospital Affiliated to Nanjing Medical University, Nanjing, China

**Keywords:** colorectal cancer (CRC), protein kinase C ζ (PKCζ), AMP-activated protein kinase (AMPK), microRNA-25-5p, cell proliferation

## Abstract

Developing novel strategies against human colorectal cancer (CRC) cells is needed. Activation of AMP-activated protein kinase (AMPK) could possibly inhibit CRC cells. Protein kinase C ζ (PKCζ) is an AMPK negative regulator. Here we found that PKCζ expression was significantly elevated in human colon cancer tissues and CRC cells. PKCζ upregulation was correlated with AMPK in-activation and mTOR complex 1 (mTORC1) over-activation. Reversely, PKCζ shRNA knockdown activated AMPK signaling and inhibited HT-29 cell proliferation. Significantly, downregulation of microRNA-25-5p (miR-25-5p), a PKCζ-targeting miRNA, could be the cause of PKCζ upregulation. Exogenous expression of miR-25-5p silenced PKCζ to activate AMPK signaling, which inhibited HT-29 cell proliferation. *In vivo* studies showed that HT-29 xenograft growth in mice was inhibited after expressing PKCζ shRNA or miR-25-5p. Collectively, PKCζ could be a novel oncogenic protein of human CRC. PKCζ silence, by targeted-shRNA or miR-25-5p expression, activates AMPK and inhibits HT-29 cell proliferation.

## INTRODUCTION

Colorectal cancer (CRC) is a major threat to human health [1, 2]. It causes large cancer motilities each year [1, 2]. With the recent progress achieved in the diagnosis and clinical treatments for CRC, the prognosis of this devastating disease has been improved in the past decades [3, 4]. Yet, for the patients with advanced, recurrent and metastatic CRC, the five-year overall survival is still poor [1, 2]. Meanwhile, the incidence of this disease is rising in China and other regions of the world [1, 2]. Therefore, it is important to indentify novel and key oncogenic proteins of human CRC.

AMP-activated protein kinase (AMPK) plays is critical in maintaining the balance of energy metabolism [5, 6]. In cancer cells, a growing body of evidences have demonstrated that this kinase is also vital for the regulation of cell survival and death [5–7]. Activation of AMPK, via modulating its downstream targeting proteins, could inhibit cancer cell proliferation and/or promote cell death [8–12]. For instance, activated AMPK could possibly induce p53 activation and mammalian target of rapamycin (mTOR) complex 1 (mTORC1) in-activation [13], as well as autophagy induction [14–17] and oncogenic protein degradation [18], which eventually can lead to profound anti-cancer cell activity. Indeed, multiple conventional chemo-drugs and natural-occurring compounds shall provoke AMPK signaling to efficiently inhibit CRC cells [8, 10, 19–22].

Recent studies have proposed protein kinase C ζ (PKCζ) as a negative regulator of AMPK [23, 24]. PKCζ was shown to phosphorylate and inactivate the AMPK kinase liver kinase B1 (LKB1), thus shutting down AMPK signaling [23, 24]. Reversely, PKCζ silence or inhibition could result in sustained AMPK activation [23, 24]. Here, we show that PKCζ upregulation in human CRC cells silences AMPK to promote cancer cell proliferation. Further, downregulation of microRNA-25-5p (“miR-25-5p”), a PKCζ-targeting miRNA [24], could be the cause of PKCζ upregulation in CRC cells.

## RESULTS

### PKCζ upregulation in human colon cancer tissues and CRC cells

**Figure 1:**
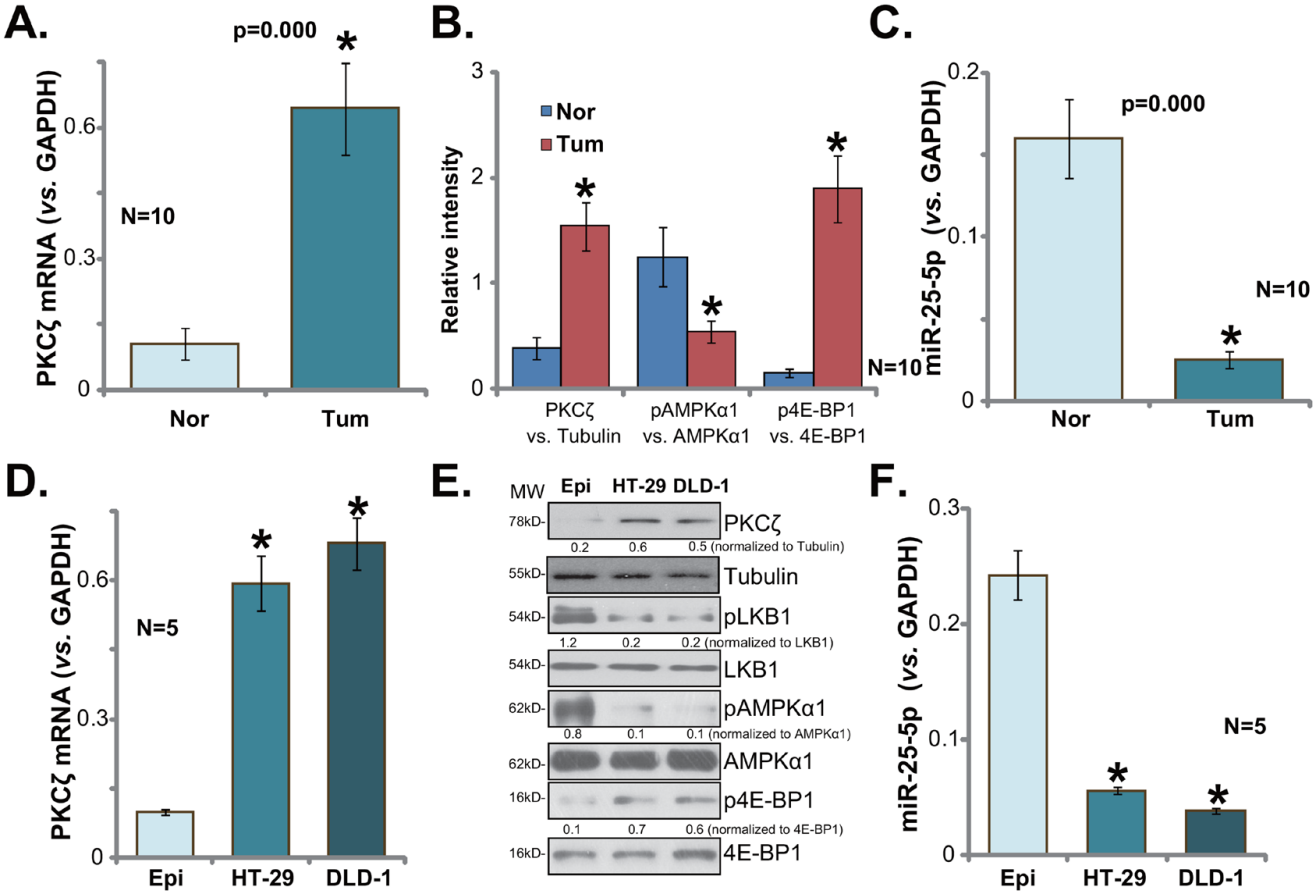
PKCζ upregulation in human colon cancer tissues and CRC cells Expressions of protein kinase C ζ (PKCζ) mRNA (**A** and **D**, qRT-PCR assay), listed proteins (**B** and **E**, Western blotting assay) and microRNA-25-5p (“miR-25-5p”, **C** and **F**, qRT-PCR assay) in fresh human colon cancer tissues (“Tum”, N=10) and surrounding normal colon tissues (“Nor”), as well as in the FHC colon epithelial cells (“Epi”) and human CRC cells (HT-29 and DLD-1) were shown. Band intensity was quantified (**B**, intergrading 10 sets of samples, and **E**). “MW” stands for molecular weight (**E**, same for all Figures). “Tubulin” stands for “β-Tubulin” (Same for all Figures). Data were expressed as mean ± SD (Same for all Figures). * *p* <0.05 *vs.* “Nor”/“Epi”.

First, we tested expression of PKCζ in human colon cancer tissues. Quantitative real-time PCR (qRT-PCR) assay was employed, and results showed that PKCζ mRNA expression level was significantly elevated in fresh colon cancer tissues (“Tum”, Figure 1A). It level was over five times higher than that in the surrounding normal colon epithelial tissues (“Nor”, Figure 1A). Quantified Western blotting assay results (integrating 10 sets of above-mentioned samples) in Figure 1B confirmed PKCζ protein upregulation in cancer tissues. As discussed, PKCζ is a negative regulator of AMPK [23, 24]. AMPK signaling was then tested in above tissues. As demonstrated, AMPK activation (p-AMPKα1 at Thr-172) was much lower in colon cancer tissues (see quantified results in Figure 1B), as compared to that in the normal tissues. AMPK activation could inhibit mTORC1 directly or indirectly [13, 25, 26]. We here showed that p-eIF4E-binding protein 1 (4E-BP1, Ser-65), indicating mTORC1 activation [27], was indeed significantly higher in the cancer tissues (See quantified results in Figure 1B). These results confirmed PKCζ upregulation in human colon cancer tissues, which was correlated with AMPK inhibition and mTORC1 activation.

A very recent study by Fan et al., has characterized a PKCζ-targeting miRNA, miR-25-5p [24]. We thus tested miR-25-5p expression in above human tissues. As demonstrated, miR-25-5p level was dramatically downregulated in colon cancer tissues (Figure 1C), and its level was relatively high in the surrounding normal tissues (Figure 1C). Expressions of above signaling proteins and miR-25-5p were also examined in human CRC cancer cells. As compared to the FHC colon epithelial cells (“Epi”) [28], PKCζ mRNA (Figure 1D) and protein (Figure 1E) expressions were both elevated in the human CRC cells (HT-29 and DLD-1), yet AMPK activation, reflected by p-LKB1 (Ser-428) and p-AMPKα1, was low (Figure 1E). Correspondingly, mTORC1 activation (p-4E-BP1) was increased in CRC cells (Figure 1E). miR-25-5p level was also decreased in the CRC cells (Figure 1F). Notably, PKCζ mRNA upregulation and miR-25-5p downregulation were also noticed in other established CRC cell lines, including HCT-116, Lovo, SW403 and SW48 (Supplementary Figure 1). Collectively, we confirmed PKCζ upregulation in human colon cancer tissues and CRC cells, which was correlated with AMPK inhibition, mTORC1 activation and miR-25-5p depletion.

### PKCζ shRNA knockdown activates AMPK and inhibits HT-29 cell proliferation

**Figure 2:**
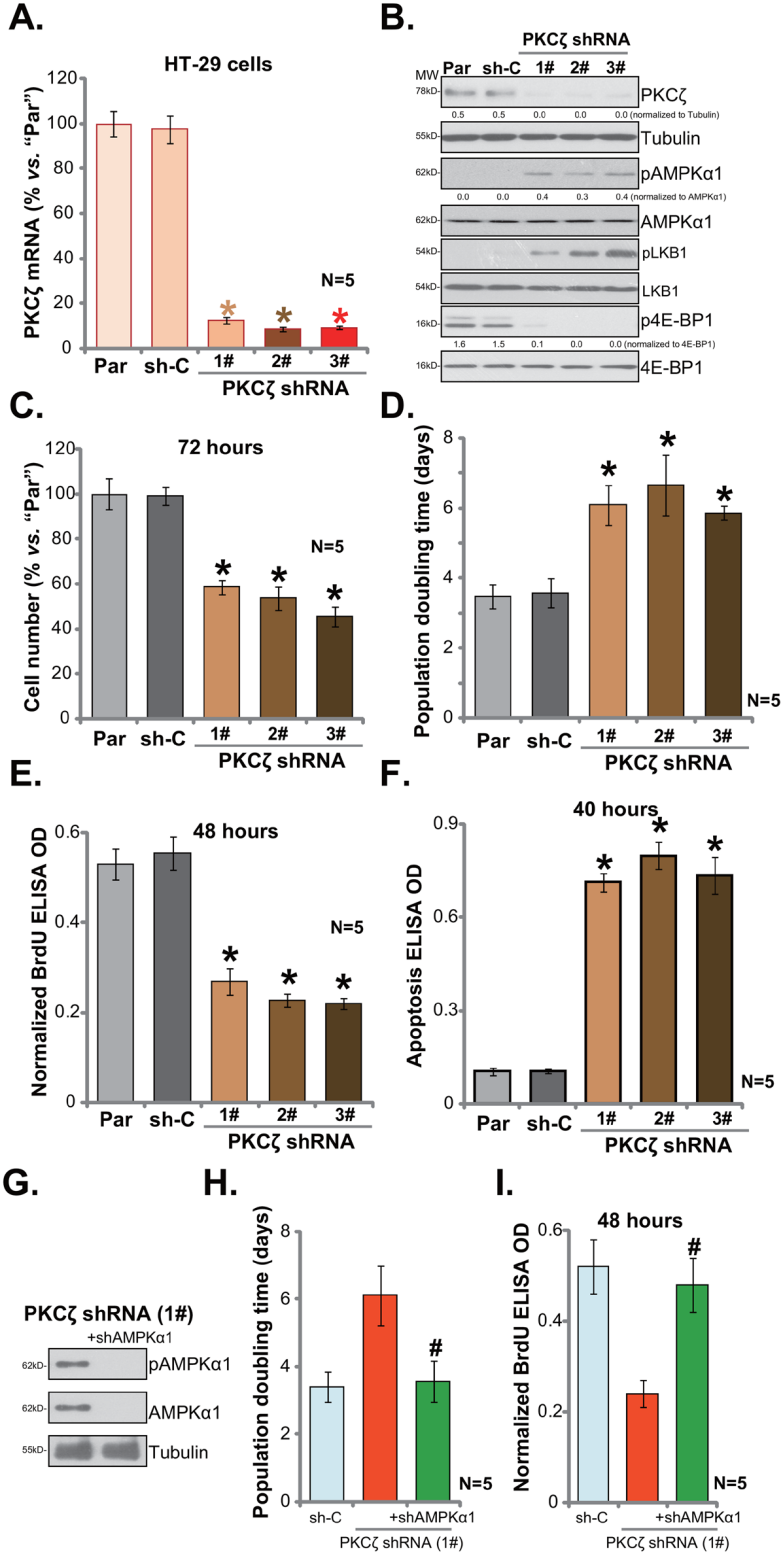
PKCζ shRNA knockdown activates AMPK and inhibits HT-29 cell proliferation Stable HT-29 cells, expressing listed PKCζ-targeting shRNA (“1#/2#/3#”), or non-sense shRNA control (“sh-C”), as well as the parental control HT-29 cells (“Par”) were subjected to qRT-PCR **(A)** assay and Western blotting assay **(B)** of listed genes; proliferation of above cells was tested by viable cell counting assay **(C)**, population doubling time was calculated in **(D)** and BrdU ELISA assay **(E)**; cell apoptosis was quantified via the Histone DNA ELISA assay **(F)**. PKCζ-targeting shRNA (“1#”)-expressing HT-29 cells were further infected with lentiviral AMPKα1 shRNA (“+shAMPKα1”), expressions of listed proteins were shown **(G)**; population doubling time **(H)** and BrdU ELISA OD **(I)** were also shown. For the proliferation and apoptosis assays, exact same number of viable cells (“trypan blue negative”) of different background was plated initially (Same for all Figures). Band intensity was quantified **(B)**. * *p* <0.05 *vs.* “sh-C”. ^#^ *p* <0.05 *vs.* “PKCζ shRNA (1#)” only group. Experiments in this figure were repeated five times, and similar results were obtained each time.

In order to study the possible function of PKCζ in CRC cells, shRNA strategy was employed. As discussed, a panel of three distant PKCζ-targeting shRNAs (“1#/2#/3#”), with non-overlapping sequences, were applied. The lentiviral shRNA was added to cultured HT-29 cells. After puromycin selection, stable cells were established. qRT-PCR assay results in Figure 2A demonstrated that PKCζ mRNA expression was indeed dramatically decreased in PKCζ shRNA-expressing stable HT-29 cells. Consequently, PKCζ protein expression was also silenced (Figure 2B). On the other hand, AMPK activation, tested again by p-AMPKα1 and p-LKB1, was increased in the PKCζ-silenced cells (Figure 2B). mTORC1 activation (p-4E-BP1) was yet inhibited (Figure 2B).

As discussed, AMPK activation could lead to mTORC1 inhibition and proliferation inhibition in CRC cells [8–10, 22]. We therefore tested proliferation of above CRC cells. Simple viable cell (trypan blue negative) counting assay results in Figure 2C demonstrated that PKCζ knockdown clearly inhibited HT-29 cell proliferation. The number of viable cells (at 72 hours) was dramatically lower in PKCζ shRNA-expressing HT-29 cells (*vs.* control parental cells, Figure 2C). Consequently, population doubling time of HT-29 cells was significantly increased after PKCζ knockdown (Figure 2D). Meanwhile, BrdU ELISA optic density (OD, normalized to the viable cell number) was also significantly decreased following PKCζ knockdown (Figure 2E). On the other hand, cell apoptosis level, tested by Histone DNA ELISA assay, was increased with PKCζ silence (Figure 2F). Notably, non-sense shRNA control (“sh-C”) failed to have above actions (Figure 2A-2F).

Next, lentiviral AMPKα1 shRNA was introduced to PKCζ shRNA(1#)-expressing HT-29 cells. Western blotting assay results demonstrated that the AMPKα1 shRNA efficiently silenced AMPKα1 in HT-29 cells (Figure 2G). Remarkably, PKCζ shRNA-caused HT-29 cell proliferation inhibition was almost completely blocked with AMPKα1 silence (Figure 2H and 2I). Population doubling time (Figure 2H) and BrdU ELISA OD (Figure 2I) were recovered with AMPKα1 knockdown. These results imply that activation of AMPK is required for PKCζ shRNA-induced HT-29 cell proliferation inhibition. Collectively, we show that PKCζ shRNA knockdown activates AMPK to inhibit HT-29 cell proliferation.

### Exogenous expression of miR-25-5p silences PKCζ and inhibits HT-29 cell proliferation

**Figure 3:**
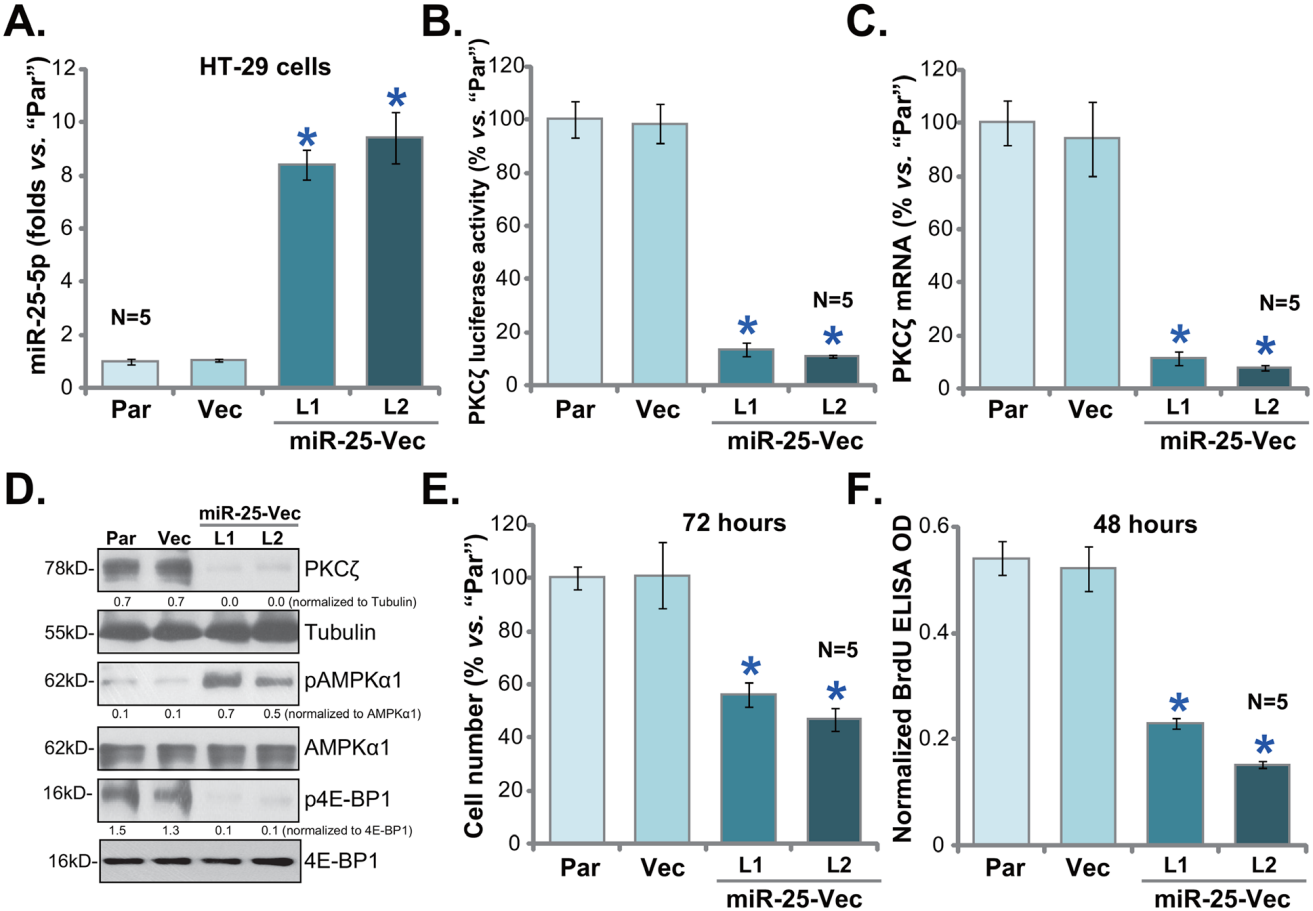
Exogenous expression of miR-25-5p silences PKCζ and inhibits HT-29 cell proliferation Stable HT-29 cells, expressing pre-miR-25-expressing vector (“miR-25-Vec”, “L1/L2”) or empty vector (“Vec”), as well as the parental control HT-29 cells (“Par”) were subjected to qRT-PCR **(A** and **C)** assay, PKCζ mRNA UTR luciferase activity assay **(B)** and Western blotting assay **(D)**; proliferation of above cells was tested by viable cell counting assay **(E)** and BrdU ELISA assay **(F)**. Band intensity was quantified **(D)**. * *p* <0.05 *vs.* “Vec”. Experiments in this figure were repeated three times, and similar results were obtained each time.

miR-25-5p is a recently-indentified PKCζ-targeting miRNA [24], its level was negatively correlated with PKCζ level in human colon cancer tissues and CRC cells (Figure 1). miR-25-5p downregulation could therefore be the cause of PKCζ upregulation. To support this hypothesis, the pre-miR-25-expressing vector (“miR-25-Vec”, a gift from Dr. Cui [24]) was introduced to the HT-29 cells. Via selection, two stable HT-29 cell lines expressing miR-25-Vec were established, named as “miR-25-Vec-L1” and “miR-25-Vec-L2”. In line with previous findings [24], expression of miR-25-5p was significantly elevated in the two lines (Figure 3A). miR-25-3p level was not changed in these cells (Data not shown), suggesting that miR-25-5p could be the primary product of the vector (reported in [24]). Exogenous expression of miR-25-5p led to a dramatic reduction of PKCζ mRNA UTR luciferase activity (Figure 3B). PKCζ mRNA (Figure 3C) and protein expression (Figure 3D) was also depleted in the two HT-29 cell lines, where AMPK activation (p-AMPKα1) and mTORC1 (p-4E-BP1) inhibition were subsequently observed (Figure 3D). As compared to the parental control cells, proliferation of the two stable cell lines was significantly inhibited, which was again tested by cell counting assay (Figure 3E) and BrdU ELISA assay (Figure 3F). Notably, the vector control (“Vec”) failed to change above gene expression and HT-29 cell proliferation (Figure 3A-3F). These results indicate that PKCζ could be the direct and primary target of miR-25-5p in HT-29 cells.

### HT-29 xenograft growth in SCID mice is inhibited after expressing PKCζ shRNA or miR-25-5p

**Figure 4:**
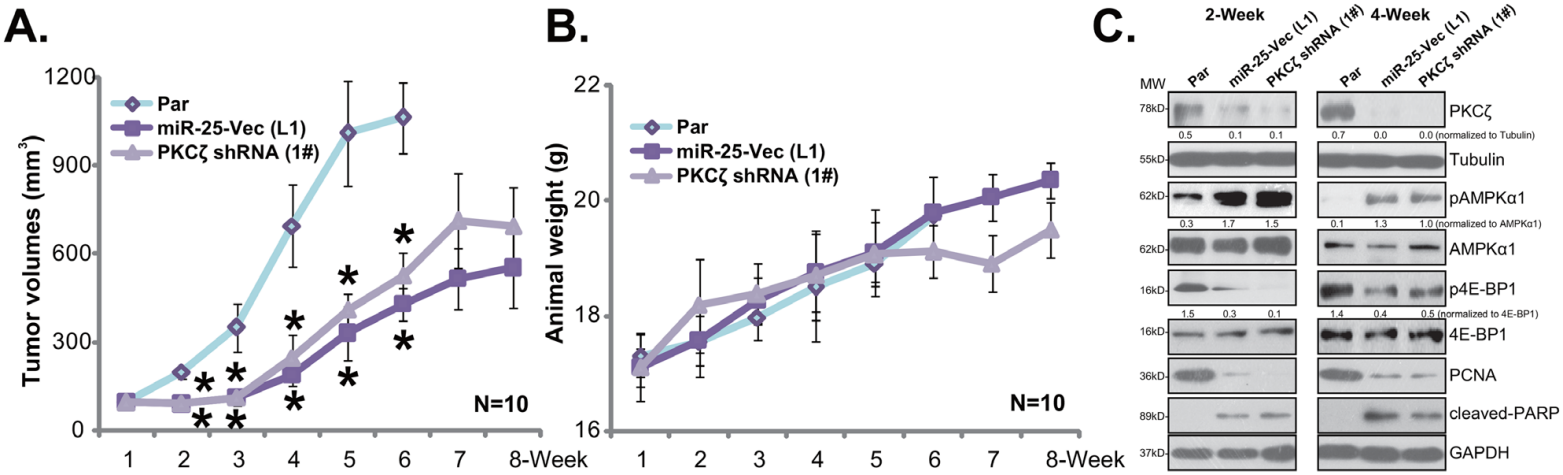
HT-29 xenograft growth in mice is inhibited after expressing PKCζ shRNA or miR-25-5p Same amount (five million cells per mouse) of HT-29 cells, bearing PKCζ shRNA (1#) or miR-25-5p (“L1”) as well as the parental control HT-29 cells (“Par”) were inoculated *s.c.* to the SCID mice. Recordings were started when the tumor volume was about 100 mm^3^ of each group. Tumor volumes **(A)** and mice body weight **(B)** were recorded weekly; at Week-2 and Week-4, one HT-29 tumor per group was isolated, expression of listed proteins in fresh tumor lysates was tested by Western blotting assay **(C)**. Band intensity was quantified **(C)**. * *p* <0.05 *vs.* “Par” tumors.

The potential effect of PKCζ shRNA or miR-25-5p on CRC cell growth *in vivo* was tested next. Same amount of HT-29 cells, bearing PKCζ shRNA (“1#”, see Figure 2), miR-25-5p (“L1”, see Figure 3) or the parental control HT-29 cells (“Par”, or control tumors) were inoculated to the SCID mice via *s.c.* injection. Tumor recordings were initiated when the tumor volume was about 100 mm^3^ of each group. Weekly tumor growth curve result in Figure 4A demonstrated that the *in vivo* growth of HT-29 xenografts was significantly inhibited after expressing the PKCζ shRNA or miR-25-5p. The tumor sizes of the two groups were much smaller than those of the control tumors (Figure 4A). Mice body weight was not significantly different between the three groups (Figure 4B). To test signaling changes in above tumor tissues, at Week-2 and Week-4, one HT-29 tumor per group was isolated. Western blotting assay was again applied to test above signaling proteins in fresh tumor lysates. As compared to the control tumors, depleted PKCζ, enhanced AMPK activation and decreased p-4E-BP1 were noticed in tumors bearing PKCζ shRNA or miR-25-5p (Figure 4C). Meanwhile, downregulation of PCNA (a proliferation marker) and induction of cleaved-PARP (an apoptosis marker) were observed in tumor tissues with PKCζ shRNA or miR-25-5p (Figure 4C) Therefore, the *in vivo* signaling changes were in line with the *in vitro* findings.

## DISCUSSION

Recent studies have implied that forced AMPK activation could be a novel and efficient strategy to inhibit CRC cells. For example, Chen et al., showed that AMPK activation mediated plumbagin-induced growth inhibition of CRC cells [9]. Kang et al., demonstrated that Widdrol-induced CRC cell death requires AMPK activation [29]. Aqueous Oldenlandia diffusa extracts and capsaicin also activated AMPK-dependent death pathway in CRC cells [8, 30]. Further, activation of AMPK-dependent autophagic pathway contributed to C6 ceramide-induced inhibition of HT-29 cells [20]. Reversely, AMPK inhibition, for example via expressing microRNA-451, could promote CRC cell proliferation and progression [10]. In the current study, we proposed that upregulation of PKCζ, the negative regulator of AMPK [23, 24], could likely lead to AMPK inhibition in human colon cancer tissues and CRC cells. On the other hand, PKCζ shRNA knockdown activated AMPK signaling and inhibited HT-29 cell proliferation *in vitro* and *in vivo*. These results imply that PKCζ could be a novel oncogenic protein for human CRC, possibly via shutting down AMPK signaling.

Interestingly, studies have also proposed that AMPK activation, under certain circumstances, could also be pro-survival [31–37] even in cancerous cells [33, 38, 39]. The difference might be due to the intensity of AMPK activation. Low to moderate AMPK activation could possibly promote cell survival, for instance via activating cytoprotective autophagy [33, 39] or limiting oxidative stress [32, 40]. Yet, sustained or intensified AMPK activation shall inhibit cancer cells via regulating above-mentioned signaling proteins (p53, mTORC1 inhibition etc.,). In fact, AMPK activity shall increase over 100-fold on phosphorylation at a conserved threonine residue (Thr-172) in the α1 subunit [41, 42]. LKB1 is indeed the AMPK α1 Thr-172 kinase [43]. PKCζ was shown to phosphorylate and inactivate LKB1, thus blocking AMPK activation [23, 24]. miR-25-5p-mediated silence of PKCζ, on the other hand, resulted in LKB1 activation and sustained/intensified AMPK activation, which should inhibit CRC cell proliferation.

miRNA-mediated gene alteration is important in controlling gene expression at the post-transcriptional level [44, 45]. miRNAs could be essential in regulating many key biologic processes of human cells, possibly via regulating expression of signaling molecules including growth factors, cytokines, transcription factors and others [46, 47]. It is known that miRNAs play vital functions in promoting CRC tumorigenesis and progression [46, 47]. miRNA dysregulation is now known as a hallmark of CRC and many other malignancies [46, 47]. In the current study, we showed that miR-25-5p, an anti-PKCζ miRNA [24], was downregulated in human colon cancer tissues and CRC cells, which could be the cause of PKCζ upregulation. Remarkably, forced exogenous expression of miR-25-5p silenced PKCζ, activated AMPK and inhibited HT-29 cell proliferation. More importantly, *in vivo* growth of HT-29 xenografts was largely suppressed after expressing miR-25-5p. These results imply that miR-25-5p could be an anti-cancer miRNA in CRC cells.

We here showed that expression of miR-25-5p inhibited CRC cell proliferation possibly via downregulating PKCζ. It is certainly possible that other targets of miR-25-5p could also be involved in above actions. For instance, several potential miR-25’s targeted genes have been identified thus far in cancer cells, including the apoptosis protein Bim [48] and mitochondrial calcium uniporter [49]. Further studies will be needed to identify possible other targets of miR-25-5p in CRC cells.

## MATERIALS AND METHODS

### Chemicals and reagents

Puromycin and neomycin were purchased from Sigma Aldrich (Shanghai, China). The reagents for cell culture were all obtained from Gibco (Shanghai, China). The PKCζ antibody was a gift from Dr. Cui [24]. All other antibodies were purchased from Santa Cruz Biotech (Santa Cruz, CA).

### Cell culture

As described, HT-29, DLD-1, HCT-116, Lovo, SW403 and SW48 human CRC cell lines were provided by Shanghai Institute of Biological Science (Shanghai, China). FHC colon epithelial cells were purchased from the iBS Fudan Cell Bank (Shanghai, China). Cells were maintained in DMEM plus 10% fetal bovine serum (FBS). The number of viable cells (trypan blue exclusive) was recorded via the TC20 automatic counter (Bio-Rad, Shanghai, China). All cell lines utilized in this study were subjected to mycoplasma and microbial contamination examination every two months. Population doubling time, colony forming efficiency, and morphology were also examined routinely for cell authentication.

### Human tissues

A total of 10 primary colon cancer patients (All polyp adenocarcinoma; 6 male and 4 females; Broders stages II-III) were enrolled in this study. The surgery-isolated colon cancer tissues and matched surrounding normal colon tissues were thoroughly washed. Tissues were then mechanically dissociated and lysed by the tissue lysis buffer (Sigma). Expressions of listed genes and miR-25-5p in fresh tissue lysates were examined. The protocols utilizing human tissue samples were in accordance with the principles expressed in the Declaration of Helsinki, and were approved by Fujian Medical University. Written-informed consent was obtained from each participant.

### BrdU assay

To test cell proliferation, cells were incubated with 10 μM of BrdU (Cell Signaling Tech, Shanghai, China). BrdU incorporation was determined in the ELISA format. BrdU OD value was always normalized to cell number.

### Histone-DNA ELISA assay

Histone-DNA ELISA PLUS kit (Roche Applied Science, Shanghai, China) was applied to quantify cell apoptosis according to the manufacturer’s protocol. ELISA OD at 450 nm was recorded.

### Quantitative real-time polymerase chain reaction (qRT-PCR) assay

In brief, total RNA was extracted by the Trizol reagents (Promega, Shanghai, China) [50]. RNA was reverse-transcribed via the SYBR Green PCR kit (Applied Biosystems, Shanghai, China). *GAPDH* primers (F-5′-AAG GTG AAG GTC GGA GTC-3′ and R-5′-TGT AGT TGA GGT CAA TGA AGG-3′) and *PKCζ* primers (F-5′-GCG TAC TGC GGC CAG TGC-3′ and R-5′-CTT GGC ATA GCT TCC ACG-3′) were provided by Dr. Cui [24]. Real-time PCR was performed using an ABI-7600 system (Shanghai, China). ^ΔΔ^Ct method was employed to quantify mRNA expression using *GAPDH* as the internal control [51, 52]. TaqMan microRNA assay system was applied to detect miR-25-5p expression using the described primer [53]. A total of 10 ng RNA per sample was reverse-transcribed using the described looped primer (Also provided by Dr. Cui [24]).

### Forced miR-25 expression

The pSuper-neo pre-miR-25 expression vector (“miR-25-Vec”) was provided by Dr. Cui [24]. The construct or the empty vector (pSuper-neo) was transfected to HT-29 cells, and stable cells were selected by neomycin (1.0 μg/mL) for 5 days. Control HT-29 cells were constructed with non-sense scramble microRNA-control (“miR-C”) [24]. Mature miR-25-5p expression in the stable cells was always tested by the qRT-PCR assay (Method was descried early [50]).

### Western blotting assay

As described [54, 55], aliquots of 30 μg (per sample) tissue or cell lysates (in RIPA lysis buffer) were electro-transferred to 10-12% SDS-PAGE gel, following by transfer to PVDF membranes. The blots were blocked by 10% milk and were then incubated with designated primary and secondary antibodies. The antigen-antibody binding was detected via enhanced chemiluminescence (ECL) reagents (GE Healthcare, Shanghai, China). The blot was quantified via ImageJ software.

### PKCζ shRNA

Three different lentiviral shRNAs (packed into the GV248-puromycin vector) against human PKCζ were reported early [24] and were provided by Genepharm Co. (Shanghai, China). HT-29 cells were seeded onto 6-well plate at 50% confluence. The lentiviral shRNA (10 μL/mL medium per well) was added to cells for 24 hours. Cells were then cultured in puromycin (1 μg/mL)-containing medium for another 5 days. PKCζ expression in the stable cells was tested by Western blotting assay and/or qRT-PCR assay. The scramble lentiviral shRNA (Genepharm Co.) was added to the control HT-29 cells.

### AMPKα1 shRNA

The AMPKα1 shRNA lentiviral particles (Santa Cruz, sc-29673-V) were added to HT-29 for 24 h. Afterwards, cell culture medium was replaced with puromycin for a total of 5 days, until resistant stable colonies were formed. AMPKα1 expression in resulting stable cells was tested by Western blotting assay.

### PKCζ mRNA luciferase assay

The UTR reporter vector that contains the 3′-UTR of PKCζ carrying the miR-25-5p site was provided again by Dr. Cui [24]. The complementary oligonucleotides for the selected region were hybridized to form double-stranded DNA and inserted into pmIR-Reporter firefly luciferase vector (Genepharm). The construct was then co-transfected with miR-25 vector into HT-29 cells. The cells were then lysed via a luciferase assay kit (Promega, Shanghai, China), which were then tested on a luminescence microplate reader.

### Xenograft assay

As described [54], the female severe combined immunodeficient (SCID) mice (age 4-5 week, weight 18-19 g) were obtained from the Animal Center of Fujian Medical University (Fuzhou, China), and were housed under standard procedures. Animals were randomized into three groups. HT-29 cells (5 × 10^6^ cells in 100 μL of saline/Matrigel, 1:1 v/v) of different genetic manipulation were inoculated subcutaneously into the flanks of the SCID mice. When the tumor reached approximately 100 mm^3^, recordings were started. The size of the tumors was measured by caliper every week, and tumor volumes were calculated using the following formula: V (volume) = 0.5328 × Long × Width × High (mm^3^) [8, 56, 57]. For recording mouse body weight, the estimated tumor weight (tumor volumes × 1g/cm^3^) was subtracted from total weight of each mouse. Mice were maintained under the following conditions: 12-hour dark/12-hour light cycle, 24 ± 2°C temperatures, and 50±10% humidity. The protocol was approved by the Nanjing Medical University’s Institutional Animal Care and Use Committee (IACUC) and Ethics Review Board (ERB).

### Statistics

The data presented were mean ± standard deviation (SD). Statistical differences were analyzed by one-way ANOVA followed by multiple comparisons with post hoc Bonferroni test (SPSS version 18.0). Values of *p* < 0.05 were considered statistically significant.

## CONCLUSION

Collectively, these results suggest that PKCζ could be a novel oncogenic protein for human CRC, possibly via shutting down AMPK signaling. On the other hand, PKCζ silence, by targeted-shRNA or miR-25-5p expression, activates AMPK and inhibits HT-29 cell proliferation.

## SUPPLEMENTARY MATERIALS FIGURE



## References

[1] Siegel RL, Miller KD, Jemal A (2016). Cancer statistics, 2016. CA Cancer J Clin.

[2] Siegel RL, Miller KD, Jemal A (2017). Cancer statistics, 2017. CA Cancer J Clin.

[3] Hubbard JM, Grothey A (2015). Colorectal cancer in 2014: progress in defining first-line and maintenance therapies. Nat Rev Clin Oncol.

[4] Palta M, Czito BG, Willett CG (2014). Colorectal cancer: adjuvant chemotherapy for rectal cancer-an unresolved issue. Nat Rev Clin Oncol.

[5] Shackelford DB, Shaw RJ (2009). The LKB1-AMPK pathway: metabolism and growth control in tumour suppression. Nat Rev Cancer.

[6] Mihaylova MM, Shaw RJ (2011). The AMPK signalling pathway coordinates cell growth, autophagy and metabolism. Nat Cell Biol.

[7] Vakana E, Altman JK, Platanias LC (2012). Targeting AMPK in the treatment of malignancies. J Cell Biochem.

[8] Lu PH, Chen MB, Ji C, Li WT, Wei MX, Wu MH (2016). Aqueous Oldenlandia diffusa extracts inhibits colorectal cancer cells via activating AMP-activated protein kinase signalings. Oncotarget.

[9] Chen MB, Zhang Y, Wei MX, Shen W, Wu XY, Yao C, Lu PH (2013). Activation of AMP-activated protein kinase (AMPK) mediates plumbagin-induced apoptosis and growth inhibition in cultured human colon cancer cells. Cell Signal.

[10] Chen MB, Wei MX, Han JY, Wu XY, Li C, Wang J, Shen W, Lu PH (2014). MicroRNA-451 regulates AMPK/mTORC1 signaling and fascin1 expression in HT-29 colorectal cancer. Cell Signal.

[11] Chen MB, Shen WX, Yang Y, Wu XY, Gu JH, Lu PH (2010). Activation of AMP-activated protein kinase is involved in vincristine-induced cell apoptosis in B16 melanoma cell. J Cell Physiol.

[12] Chen MB, Jiang Q, Liu YY, Zhang Y, He BS, Wei MX, Lu JW, Ji Y, Lu PH (2015). C6 ceramide dramatically increases vincristine sensitivity both *in vivo* and *in vitro*, involving AMP-activated protein kinase-p53 signaling. Carcinogenesis.

[13] Inoki K, Zhu T, Guan KL (2003). TSC2 mediates cellular energy response to control cell growth and survival. Cell.

[14] Kim J, Kundu M, Viollet B, Guan KL (2011). AMPK and mTOR regulate autophagy through direct phosphorylation of Ulk1. Nat Cell Biol.

[15] Egan DF, Shackelford DB, Mihaylova MM, Gelino S, Kohnz RA, Mair W, Vasquez DS, Joshi A, Gwinn DM, Taylor R, Asara JM, Fitzpatrick J, Dillin A (2011). Phosphorylation of ULK1 (hATG1) by AMP-activated protein kinase connects energy sensing to mitophagy. Science.

[16] Liang P, Le W (2015). Role of autophagy in the pathogenesis of multiple sclerosis. Neurosci Bull.

[17] Guo D, Ying Z, Wang H, Chen D, Gao F, Ren H, Wang G (2015). Regulation of autophagic flux by CHIP. Neurosci Bull.

[18] Wu WD, Hu ZM, Shang MJ, Zhao DJ, Zhang CW, Hong DF, Huang DS (2014). Cordycepin down-regulates multiple drug resistant (MDR)/HIF-1alpha through regulating AMPK/mTORC1 signaling in GBC-SD gallbladder cancer cells. Int J Mol Sci.

[19] Sugiyama M, Takahashi H, Hosono K, Endo H, Kato S, Yoneda K, Nozaki Y, Fujita K, Yoneda M, Wada K, Nakagama H, Nakajima A (2009). Adiponectin inhibits colorectal cancer cell growth through the AMPK/mTOR pathway. Int J Oncol.

[20] Huo HZ, Wang B, Qin J, Guo SY, Liu WY, Gu Y (2013). AMP-activated protein kinase (AMPK)/Ulk1-dependent autophagic pathway contributes to C6 ceramide-induced cytotoxic effects in cultured colorectal cancer HT-29 cells. Mol Cell Biochem.

[21] Hsu YF, Sheu JR, Lin CH, Yang DS, Hsiao G, Ou G, Chiu PT, Huang YH, Kuo WH, Hsu MJ (2012). Trichostatin A and sirtinol suppressed survivin expression through AMPK and p38MAPK in HT29 colon cancer cells. Biochim Biophys Acta.

[22] Din FV, Valanciute A, Houde VP, Zibrova D, Green KA, Sakamoto K, Alessi DR, Dunlop MG (2012). Aspirin inhibits mTOR signaling, activates AMP-activated protein kinase, and induces autophagy in colorectal cancer cells. Gastroenterology.

[23] Deepa SS, Zhou L, Ryu J, Wang C, Mao X, Li C, Zhang N, Musi N, DeFronzo RA, Liu F, Dong LQ (2011). APPL1 mediates adiponectin-induced LKB1 cytosolic localization through the PP2A-PKCzeta signaling pathway. Mol Endocrinol.

[24] Fan JB, Liu W, Zhu XH, Yi H, Cui SY, Zhao JN, Cui ZM (2017). microRNA-25 targets PKCzeta and protects osteoblastic cells from dexamethasone via activating AMPK signaling. Oncotarget.

[25] Gwinn DM, Shackelford DB, Egan DF, Mihaylova MM, Mery A, Vasquez DS, Turk BE, Shaw RJ (2008). AMPK phosphorylation of raptor mediates a metabolic checkpoint. Mol Cell.

[26] Hardie DG (2008). AMPK and Raptor: matching cell growth to energy supply. Mol Cell.

[27] Dancey J (2010). mTOR signaling and drug development in cancer. Nat Rev Clin Oncol.

[28] Xiang S, Wang N, Hui P, Ma J (2017). Gab3 is required for human colorectal cancer cell proliferation. Biochem Biophys Res Commun.

[29] Kang MR, Park SK, Lee CW, Cho IJ, Jo YN, Yang JW, Kim JA, Yun J, Lee KH, Kwon HJ, Kim BW, Lee K, Kang JS (2012). Widdrol induces apoptosis via activation of AMP-activated protein kinase in colon cancer cells. Oncol Rep.

[30] Kim YM, Hwang JT, Kwak DW, Lee YK, Park OJ (2007). Involvement of AMPK signaling cascade in capsaicin-induced apoptosis of HT-29 colon cancer cells. Ann N Y Acad Sci.

[31] Narbonne P, Roy R (2009). Caenorhabditis elegans dauers need LKB1/AMPK to ration lipid reserves and ensure long-term survival. Nature.

[32] Jeon SM, Chandel NS, Hay N (2012). AMPK regulates NADPH homeostasis to promote tumour cell survival during energy stress. Nature.

[33] Min H, Xu M, Chen ZR, Zhou JD, Huang M, Zheng Zou XP (2014). Bortezomib induces protective autophagy through AMP-activated protein kinase activation in cultured pancreatic and colorectal cancer cells. Cancer Chemother Pharmacol.

[34] Lv G, Zhu H, Zhou F, Lin Z, Lin G, Li C (2014). AMP-activated protein kinase activation protects gastric epithelial cells from Helicobacter pylori-induced apoptosis. Biochem Biophys Res Commun.

[35] Accordi B, Galla L, Milani G, Curtarello M, Serafin V, Lissandron V, Viola G, te Kronnie G, De Maria R, Petricoin EF, Liotta LA, Indraccolo S, Basso G (2013). AMPK inhibition enhances apoptosis in MLL-rearranged pediatric B-acute lymphoblastic leukemia cells. Leukemia.

[36] Liu W, Mao L, Ji F, Chen F, Hao Y, Liu G (2017). Targeted activation of AMPK by GSK621 ameliorates H2O2-induced damages in osteoblasts. Oncotarget.

[37] Herrero-Martin G, Hoyer-Hansen M, Garcia-Garcia C, Fumarola C, Farkas T, Lopez-Rivas A, Jaattela M (2009). TAK1 activates AMPK-dependent cytoprotective autophagy in TRAIL-treated epithelial cells. EMBO J.

[38] Zhou C, Gu J, Zhang G, Dong D, Yang Q, Chen MB, Xu D (2017). AMPK-autophagy inhibition sensitizes icaritin-induced anti-colorectal cancer cell activity. Oncotarget.

[39] Zhu LQ, Zhen YF, Zhang Y, Guo ZX, Dai J, Wang XD (2013). Salinomycin activates AMP-activated protein kinase-dependent autophagy in cultured osteoblastoma cells: a negative regulator against cell apoptosis. PLoS One.

[40] She C, Zhu LQ, Zhen YF, Wang XD, Dong QR (2014). Activation of AMPK protects against hydrogen peroxide-induced osteoblast apoptosis through autophagy induction and NADPH maintenance: new implications for osteonecrosis treatment?. Cell Signal.

[41] Hardie DG, Ross FA, Hawley SA (2012). AMPK: a nutrient and energy sensor that maintains energy homeostasis. Nat Rev Mol Cell Biol.

[42] Hardie DG, Ross FA, Hawley SA (2012). AMP-activated protein kinase: a target for drugs both ancient and modern. Chem Biol.

[43] Shaw RJ, Kosmatka M, Bardeesy N, Hurley RL, Witters LA, DePinho RA, Cantley LC (2004). The tumor suppressor LKB1 kinase directly activates AMP-activated kinase and regulates apoptosis in response to energy stress. Proc Natl Acad Sci U S A.

[44] Lu J, Getz G, Miska EA, Alvarez-Saavedra E, Lamb J, Peck D, Sweet-Cordero A, Ebert BL, Mak RH, Ferrando AA, Downing JR, Jacks T, Horvitz HR (2005). MicroRNA expression profiles classify human cancers. Nature.

[45] Calin GA, Croce CM (2006). MicroRNA signatures in human cancers. Nat Rev Cancer.

[46] Orang AV, Barzegari A (2014). MicroRNAs in colorectal cancer: from diagnosis to targeted therapy. Asian Pac J Cancer Prev.

[47] Muhammad S, Kaur K, Huang R, Zhang Q, Kaur P, Yazdani HO, Bilal MU, Zheng J, Zheng L, Wang XS (2014). MicroRNAs in colorectal cancer: role in metastasis and clinical perspectives. World J Gastroenterol.

[48] Zhang H, Zuo Z, Lu X, Wang L, Wang H, Zhu Z (2012). MiR-25 regulates apoptosis by targeting Bim in human ovarian cancer. Oncol Rep.

[49] Marchi S, Lupini L, Patergnani S, Rimessi A, Missiroli S, Bonora M, Bononi A, Corra F, Giorgi C, De Marchi E, Poletti F, Gafa R, Lanza G (2013). Downregulation of the mitochondrial calcium uniporter by cancer-related miR-25. Curr Biol.

[50] Jia X, Wang F, Han Y, Geng X, Li M, Shi Y, Lu L, Chen Y (2016). miR-137 and miR-491 negatively regulate dopamine transporter expression and function in neural cells. Neurosci Bull.

[51] Li ZW, Cai S, Liu Y, Yang CL, Tian Y, Chen G, Cao C (2016 Aug 1). Over-expression of Galphai3 in human glioma is required for Akt-mTOR activation and cell growth. Oncotarget.

[52] Gong YQ, Huang W, Li KR, Liu YY, Cao GF, Cao C, Jiang Q (2016). SC79 protects retinal pigment epithelium cells from UV radiation via activating Akt-Nrf2 signaling. Oncotarget.

[53] Gao W, Chan JY, Wong TS (2014). Curcumin exerts inhibitory effects on undifferentiated nasopharyngeal carcinoma by inhibiting the expression of miR-125a-5p. Clin Sci (Lond).

[54] Zhang S, Deng Z, Yao C, Huang P, Zhang Y, Cao S, Li X (2017). AT7867 inhibits human colorectal cancer cells via AKT-dependent and AKT-independent mechanisms. PLoS One.

[55] Wang HC, Zhang T, Kuerban B, Jin YL, Le W, Hara H, Fan DS, Wang YJ, Tabira T, Chui DH (2015). Autophagy is involved in oral rAAV/Abeta vaccine-induced Abeta clearance in APP/PS1 transgenic mice. Neurosci Bull.

[56] Lu XS, Qiao YB, Li Y, Yang B, Chen MB, Xing CG (2017). Preclinical study of cinobufagin as a promising anti-colorectal cancer agent. Oncotarget.

[57] Chen MB, Zhou ZT, Yang L, Wei MX, Tang M, Ruan TY, Xu JY, Zhou XZ, Chen G, Lu PH (2016). KU-0060648 inhibits hepatocellular carcinoma cells through DNA-PKcs-dependent and DNA-PKcs-independent mechanisms. Oncotarget.

