# Supplementary material for: Silencing protein kinase C ζ by microRNA-25-5p activates AMPK signaling and inhibits colorectal cancer cell proliferation
Source: Oncotarget. 2017 Jun 27;8(39):65329–38. doi: 10.18632/oncotarget.18649 (PMC5630334; doi:10.18632/oncotarget.18649)
Supplement: Supplementary file 1 [file oncotarget-08-65329-s001.pdf]

Silencing protein kinase C ζ by microRNA-25-5p activates AMPK signaling and inhibits colorectal cancer cell proliferation

SUPPLEMENTARY MATERIAL

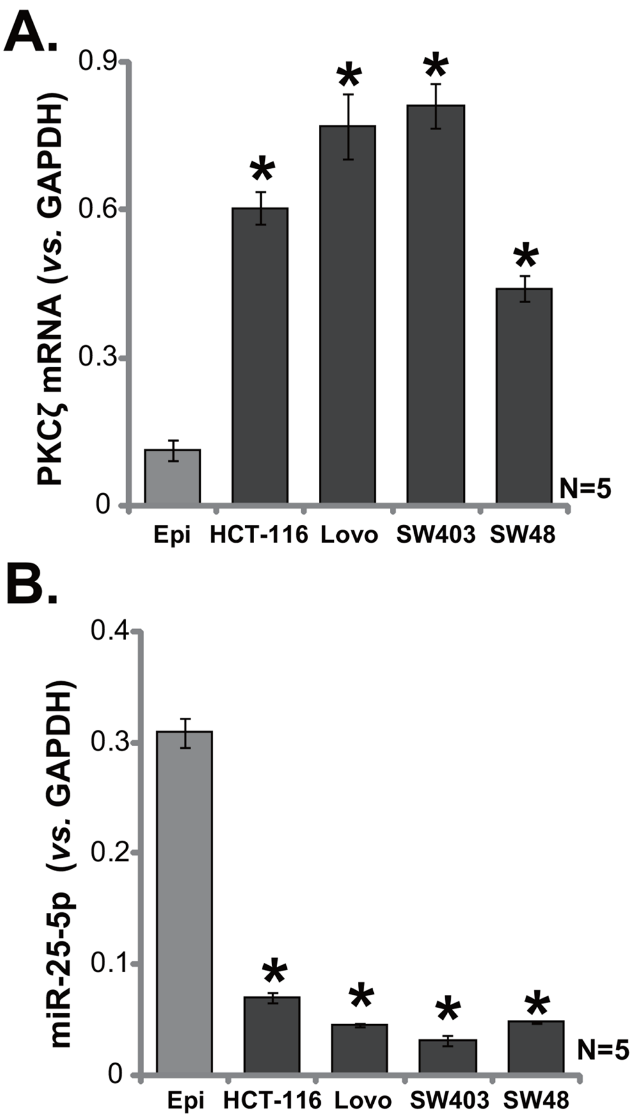

Supplementary Figure 1: Expressions of protein kinase C ζ (PKCζ) mRNA (A), and microRNA-25-5p ("miR-25-5p", B) in FHC colon epithelial cells ("Epi") and human CRC cells (HCT-116, Lovo, SW403 and SW48) were tested by qRT-PCR assay. * p <0.05 *vs.* "Nor"/"Epi". Experiments in this figure were repeated five times, and similar results were obtained each time.
